# Homeobox Genes and Hepatocellular Carcinoma

**DOI:** 10.3390/cancers11050621

**Published:** 2019-05-03

**Authors:** Kwei-Yan Liu, Li-Ting Wang, Shih-Hsien Hsu, Shen-Nien Wang

**Affiliations:** 1 Graduate Institute of Medicine, College of Medicine, Koahiusng 807, Taiwan; a52t5213b@hotmail.com (K.-Y.L.); innywang91104@gmail.com (L.-T.W.); 2 Department of Medical Research, Kaohsiung Medical University Hospital, Koahiusng 807, Taiwan; 3 Division of Hepatobiliary Surgery, Department of Surgery, Kaohsiung Medical University Hospital, Koahiusng 807, Taiwan; 4 Department of Surgery, Faculty of Medicine, Kaohsiung Medical University, Koahiusng 807, Taiwan

**Keywords:** Homeobox, HCC, EMT, immunosuppression, IL6

## Abstract

Hepatocellular carcinoma (HCC) is the sixth most common type of cancer, and is the third leading cause of cancer-related deaths each year. It involves a multi-step progression and is strongly associated with chronic inflammation induced by the intake of environmental toxins and/or viral infections (i.e., hepatitis B and C viruses). Although several genetic dysregulations are considered to be involved in disease progression, the detailed regulatory mechanisms are not well defined. Homeobox genes that encode transcription factors with homeodomains control cell growth, differentiation, and morphogenesis in embryonic development. Recently, more aberrant expressions of Homeobox genes were found in a wide variety of human cancer, including HCC. In this review, we summarize the currently available evidence related to the role of Homeobox genes in the development of HCC. The objective is to determine the roles of this conserved transcription factor family and its potential use as a therapeutic target in future investigations.

## 1. Epidemiology

Hepatocellular carcinoma (HCC) is the most common type of primary liver cancer and is ranked as the second leading cause of cancer-related mortality worldwide [1]. The global annual death as a result of HCC was reported to be 700,000 patients [2]. Intriguingly, its incidence worldwide differs from heterogeneous prevalence of risk factors. The highest incidence of HCC has been reported in East/Southeast Asia and Africa and the lowest in South/Central Asia and Europe [3]. Commonly mentioned risk factors for HCC include chronic viral hepatitis (i.e., hepatitis B virus (HBV) and hepatitis C virus (HCV)), chronic alcohol use, environment pollutants, obesity, and diabetes mellitus. Chronic hepatitis B and exposure to aflatoxin are major risk factors for the occurrence of HCC in sub-Saharan Africa and East Asia, whereas chronic hepatitis C is the major risk factor in the USA, Europe, and Japan [4]. In recent years, nonalcoholic fatty liver disease (NAFLD) has gradually emerged as a leading cause of HCC in Western and Asian populations [5].

## 2. Etiologies

Various possible mechanisms, which link these risk factors to hepatocarcinogenesis, have been proposed. HBV—an enveloped DNA virus—belongs to the *Hepadnaviridae* family and includes eight genotypes (i.e., A to H), which have their respective geographical distribution [6]. Studies have shown that the HBV X protein (HBx) is a 154-amino acid polypeptide that plays an essential role in the development of HCC. HBx may directly promote hepatocytes transforming into tumor-initiating cells through the activation of Wnt/β-catenin signaling [7]. HCV is a single-stranded positive RNA virus belonging to the *Hepacivirus* genus in the family Flavivaridae, including seven major genotypes [8]. HCV-induced progressive liver cirrhosis is a well-known risk factor for the development of HCC. Of note, HCC can occur more than 10 years after eradication of HCV, with an annual rate of 1% [9]. Liver cirrhosis is an established risk factor for HCC; it represents the final stage of liver fibrosis and usually develops in response to chronic liver injuries [10]. Chronic alcohol consumption and consequent liver cirrhosis play a causative role in the development of HCC. The consumption of contaminated animal and plant products may expose individuals to aflatoxins, another common risk factor for the development of HCC. Aflatoxin B1 (AFB1) is the most potent liver carcinogen among the four aflatoxins (i.e., B1, B2, G1, and G2). *p53* gene mutations are associated with high exposure to AFB1. These mutations, such as codon 249 transversion, appeared in 50% of HCC cases [11]. NAFLD encompasses a spectrum of pathological changes characterized by different degrees of fat accumulation in the hepatocytes. This condition is attributable to overnutrition and is strongly associated with metabolic syndrome. Nonalcoholic steatohepatitis (NASH) is a severe subtype of NAFLD, with the histologic features of lobular inflammation and hepatocyte ballooning. Patients with NASH are predisposed to liver fibrosis, cirrhosis, and HCC [12]. Several mechanisms, including increased levels of tumor necrosis factor-α (TNF-α), interleukin 6 (IL-6), and leptin, have been correlated with carcinogenesis from NASH.

## 3. Treatments

In the past, HCC was usually diagnosed at an advanced stage, following the development of symptoms and impairment of liver function. At that point, treatment was often futile with poor median survival rates (i.e., <3 months) [13]. Currently, a substantial proportion of HCC patients continues to have a poor liver reserve and/or compromised portal vein flow. Thus, these untreated cases of HCC are associated with poor prognosis. With advancements in early HCC detection technology and surveillance programs, the curative treatment has improved the five-year survival rates, ranging from 50% to 75% [14]. Despite the availability of several therapeutic options for HCC (i.e., hepatic resection, liver transplantation, locoregional therapies, and systemic therapies), the treatment strategy must be individualized for each patient. The Barcelona Clinic Liver Cancer staging system is widely used worldwide to establish the prognosis and most appropriate treatment strategy for patients at different stages [15]. Although the so-called curative treatments (i.e., surgical resection, liver transplantation, and radiofrequency ablation) have greatly improved the outcomes of HCC, disease recurrence and intrahepatic metastasis continue to pose challenges in the treatment of these patients. The Barcelona Clinic Liver Cancer algorithm suggests systemic treatment for advanced HCC. Sorafenib—an oral multikinase inhibitor of cell proliferation through a strong inhibition of the serine/threonine kinase RAF—is the first approved systemic medication for the treatment of advanced HCC [16,17]. The efficacy of sorafenib has been demonstrated in several clinical studies [18]. However, although sorafenib is currently considered the best option for treating advanced HCC, it only increases life expectancy by a few months. In the recent years, certain limitations of radiotherapy for HCC treatment, such as inability to deliver a tumoricidal dose, have been overcome. In patients who are not candidates for orthotopic liver transplantation or resection, the tumor can be precisely targeted to deliver the appropriate dose through modern liver-directed radiotherapy, including three-dimensional conformal radiotherapy, charged particle radiotherapy, and stereotactic body radiotherapy [19]. HCC develops several immunosuppressive mechanisms to evade the immunological surveillance system and progress further. The immune checkpoint regulation and its associated molecules have led to advancements in cancer treatment. Among the different types of molecules involved in the immune checkpoints, programmed death-ligand 1 (PD-L1), found on the surface of cancer cells and stromal cells; programmed cell death 1 (PD-1); and cytotoxic T lymphocyte-associated protein 4, found on the surface of T cells have been shown to participate in the crucial steps for the suppression of T-cell function by cancer cells [20,21]. Active efforts toward immunotherapy for HCC include the development of monoclonal antibodies against molecules of the immune checkpoint [22].

## 4. Homeobox Genes

**Figure 1:**
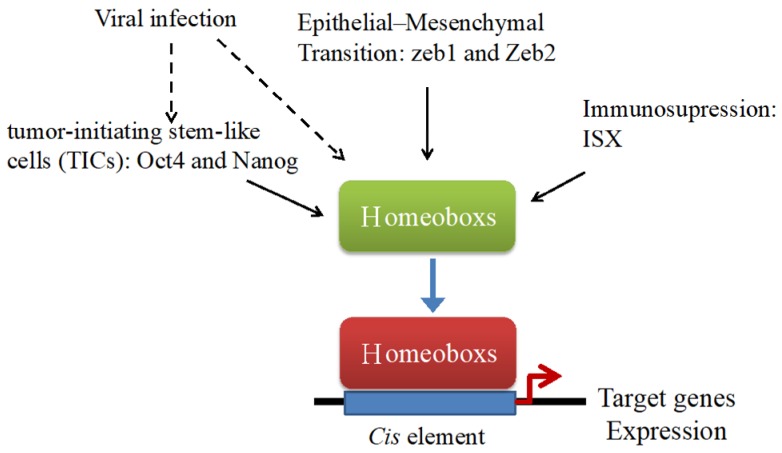
Homeobox genes participate in the generation of hepatocellular carcinoma (HCC). The Homeobox genes positively and negatively regulate in tumorigenesis of HCC, including tumor-initiating stem-like cells, epithelial to mesenchymal transition, immunotolerance, and viral infection (hepatitis B virus (HBV) and hepatitis C virus (HCV)). The altered transcriptional regulation of Homeobox genes that we assigned in the figure dramatically rearranges the downstream genes’ expression, which participates in promotion of HCC tumorigenesis.

Homeobox genes, which are master regulatory genes controlling the development of each segment, were firstly discovered in the fruit fly *Drosophila melanogaster* [23]. In flies, three Homeobox genes (*Ubx*, *Abd-A*, and *Abd-B*) belong to the bithorax complex and the other five Homeobox genes (*Lab*, *Pb*, *Dfd*, *Scr*, and *Antp*) belong to the antennapedia complex. Homeobox genes specify the regional identity from the anterior to the posterior of the body segments of the fly [24]. The concept that a cluster of regulatory genes may control the development of segments is conserved in a wide range of organisms (from *Caenorhabditis elegans* to humans) [25]. In humans, the Homeobox genes are classified into four clusters (i.e., A to D) and located at 7p15, 17q21.2, 12q13, and 2q31. On the basis of sequence similarity and relative position of the cluster, each cluster is identified with 13 paralog groups with 9–11 protein-coding genes [26]. The Homeobox gene has a consensus element with 180 bps, and its sequence encodes approximately 60 amino acids conserved homeodomain, which is a DNA-binding globular domain. This domain contains a stable three-helix bundle preceded by a flexible N-terminal arm [27]. On the basis of the evolutionary tree, Homeobox genes can be classified into 11 classes (i.e., ANTP, PRD, LIM, POU, HF, SINE, TALE, CUT, PROS, ZF, and CERS), containing approximately 250 Homeobox genes in humans [26]. The increasing numbers of Homeobox genes are as a result of two extra rounds of genome duplication, and subsequent loss of paralogs has been discovered in humans [23]. The Homeobox genes are involved in various processes ranging from the earliest stages of development, embryonic stem cells [28], to patterning (particularly the *Homeobox* genes). Notably, mutations in the Homeobox genes cause developmental defects [29]. An increasing number of studies have demonstrated the aberrant expression of Homeobox genes in tumorigenesis, suggesting that, besides developmental regulation, these genes play a critical role in the development of cancer [30]. In this review, we will focus on the role of the Homeobox genes in the generation of HCC, including tumor-initiating stem-like cells (TICs), epithelial to mesenchymal transition (EMT), immunotolerance, and viral infection (Figure 1), and list the promoting or repressing function of Homeobox genes in HCC (Table 1 and Table 2).

## 5. Homeobox Genes (*Oct4 and Nanog*) in TICs of HCC

TICs have been known to be capable of recapitulating the primary tumor heterogeneity owing to their self-renewal, differentiation, and tumor-initiation capacities [98,99]. One of the main properties of TICs is their self-renewal ability, which has been identified through performing sphere formation assays in vitro and through transplantation experiments with limiting dilution in mice [100,101]. Several cell-surface antigens have been discovered on liver TICs, including PROM1 (CD133), THY1 (CD90), epithelial cell adhesion molecule (EpCAM), and CD24 [102]. Increasing evidence indicates that liver TICs play a critical role in hepatocarcinogenesis [66,103]. In Homeobox genes, octamer-binding transcription factor 4 (*Oct4*) and *Nanog* reportedly control the self-renewal and pluripotency of pluripotent stem cells [104,105]. Moreover, both of them were identified as critical non-cell-surface markers in TICs of various types of tumors [106,107]. Therefore, it is essential to investigate the mechanism through which the Homeobox genes are regulated in TICs. The elucidation of this mechanism may assist treating physicians in achieving improved clinical outcomes for patients with HCC. *MiR-429* has been identified as an oncogene in HCC by promoting the self-renewal, tumorigenicity, and chemoresistance of EpCAM-positive TICs. *MiR-429* promotes the transcription of *Oct4* by reducing the expression of retinoblastoma-binding protein 4, which otherwise inhibits the *E2F1* transactivation of *Oct4* transcription [68]. The HCC cells with high *Nanog* expression, which have stem-cell-like characteristics, are characterized by a high capacity for tumor invasion and metastasis and are resistant to treatment with sorafenib and cisplatin [62]. These cells are required for *Nanog* to promote the expression of insulin-like growth factor (IGF) 2 and IGF 1 receptor (IGF1R) for self-renewal [62], and the NODAL/SMAD3 signaling pathway for tumor invasion [63]. It has been reported that EMT-associated transcription factors Snail and Slug or the androgen/androgen receptor axis directly regulate the expression of *Nanog* under transforming growth factor-β (TGF-β) signaling in TICs [58,59,64]. Interestingly, chromatin immunoprecipitation-sequencing analyses of *Nanog* showed that it regulates the expression of mitochondrial metabolic genes required in CD133-positive TICs. Moreover, it represses mitochondrial oxidative phosphorylation genes to prevent the reactive oxygen species (ROS) induction and induce oxidation of fatty acid for self-renewal and drug resistance of TICs [66]. Nevertheless, in murine nonalcoholic steatohepatitis model, *Nanog* contribute to the reprogramming of hepatic progenitor cells in driving these cells to TICs [60].

Increasing studies have also focused on signaling pathways that can manipulate the *Nanog* expression to alter the properties of TICs. Receptor for activated C kinase1 (RACK1) was first discovered through interactions between the activated form of protein kinase C, which positively regulates self-renewal and chemoresistance of CD13, and CD133 double-positive HCC cells by directly binding with *Nanog*. Its binding abrogated the recruitment of E3 ubiquitin ligase FBXW8 and degradation of proteasome to promote stemness and chemoresistance [108]. Myeloid cell leukemia-1 (Mcl-1) promotes HCC stemness and self-renewal by regulating the level of Nanog, Sox2, and KLF4 [109]. Transforming acidic coiled-coil protein 3 (TACC3), which is involved in cell mitosis and transcriptional activity, is required for Wnt/β-catenin and PI3K/AKT signaling pathways to maintain stem cell transcription factors, including Bmi1, c-Myc, and *Nanog* [110]. In addition, negative regulators of *Nanog* expression in TICs have been identified; for example, the GATA transcription factor 5 (GATA5) negatively regulates the expression of Oct4, *Nanog*, Klf4, c-myc, and EpCAM in TICs by binding with β-catenin and inhibiting β-catenin nuclear translocation [111]. Levels of Atonal homolog 8 (ATOH8), a basic-helix-loop-helix (bHLH) transcription factor, are reduced in HCC patients, and loss of ATOH8 increases the transcription of OCT4, NANOG, and CD133 and reduces chemo-sensitivity to 5-fluorouracil and cisplatin [112].

## 6. Homeobox in EMT of HCC

EMT is described as a conversion process of adherent epithelial cells into migratory mesenchymal cells with highly invasive properties. In HCC, EMT are endowed with HCC progression by inhibiting apoptosis and senescence, escaping immune reactions, and gaining chemoresistance [113,114,115], and are also involved in the early stages of tumor transformation into aggressive malignancies by increasing the potential for invasiveness and metastasis. Typically, cells undergoing EMT are characterized by decreased levels of E-cadherin, increased levels of N-cadherin and vimentin, and translocation of β-catenin from the membrane to the nucleus. An increasing number of studies have shown that Homeobox genes can positively or negatively regulate EMT in HCC as follows.

Zinc finger E-box binding Hox 1 (ZEB1) and ZEB2 are master transcription factors that positively regulate invasion and metastasis by promoting EMT in cancer cells [116]. Both ZEB1 and ZEB2 comprise two zinc finger domains at the N- and C-terminals and the Smad interaction domain, homeodomain, and CtBP interaction domain at the central region. In breast cancer, ZEB1 and ZEB2 have been shown to directly bind to the E-box located in the E-cadherin promoter [117], recruiting the CtBP transcriptional co-repressors [118] and/or the SWI/SNF chromatin remodeling protein BRG1 [119]. This results in repression of E-cadherin expression and promotion of EMT. In HCC patients, ZEB1 expression has been associated with low expression of E-cadherin, venous invasion, and tumor/node/metastasis (TNM)stage. Patients expressing high levels of ZEB1 and low levels of E-cadherin are associated with poorer prognosis [78].

In recent years, several regulations have been identified upstream of ZEB1 or ZEB2. Depletion of thrombomodulin, a natural anticoagulation factor, induces HCC cell migration by induction of ZEB1 and reduction of E-cadherin [76]. Moreover, 14-3-3e—a protein belonging to the highly conserved in eukaryotic cells 14-3-3 protein family—suppresses the expression of E-cadherin via regulation of ZEB1 [77]. It has been shown that this regulation increases the risk of metastasis and decreases the survival rates in HCC patients. Previous studies have shown that MYC-associated zinc finger protein (MAZ) promotes the expression of *c-Myc*, *Ras*, *VEGF*, and podoplanin and represses that of *p53*, *Sp4*, and endothelial nitric oxide synthase in tumor development. MAZ was also shown to promote EMT and metastasis in HCC patients [85]. Depletion of the minus-end-directed motor protein kinesin family member C1 [87]—also termed HSET—downregulates ZEB1 to reduce EMT in HCC. Numerous studies have shown that mutations of the liver kinase B1 cause cancer (i.e., lung, mammary gland, ovarian, melanoma, and HCC). In addition, it is involved in liver carcinogenesis by promoting ZEB1 expression-associated EMT [89]. Forkhead box Q1, a forkhead transcription factor, induces EMT in HCC by up-regulating the expression of ZEB2 [92].

Regulatory proteins can manipulate the levels of ZEB1 and ZEB2 in EMT. However, there are numerous microRNAs (miRNAs) and long non-coding RNAs (lncRNAs) that participate in ZEB1-mediated EMT [120,121]. For example, miR-139-5p, which suppresses EMT in HCC by binding to ZEB1 and ZEB2 mRNA, was downregulated in HCC tissue [94]. In addition, MiR-101 binds to the 3′-untranslated region of ZEB1 to silence and disrupt the EMT in HCC. However, this regulation can be reversed by small nucleolar host gene 6 transcript, acting as a competing endogenous RNA [81,84]. MiR-211-5p and miR-154 targeting ZEB2 suppress HCC metastasis and tumor growth [93,97]. *Gα12 gep* is an oncogene that generates G-protein-coupled receptors sensing the increasing levels of ligands in tumor microenvironments. The activated *Gα12* promotes EMT through the induction of ZEB1 and reduction of miRNAs (i.e., miR-192, miR-215, and miR-200a), which target ZEB1 and ZEB2 [80,90]. Long non-coding RNA activated by TGF-β (lncRNA-ATB) promotes EMT under TGF-β stimulation by binding with the miR-200 family, which directly targets ZEB1 and ZEB2 mRNA [79]. Another lncRNA ZFAS1 also up-regulated the expression of ZEB1 by binding competitively to miR-150, consequently inducing EMT and invasion [82]. An increasing number of studies have shown that upstream antisense transcription controls the transcription of the corresponding genes [122]. Interestingly, a non-coding antisense transcript, ZEB1 antisense 1, is located in physical contiguity with ZEB1. ZEB1 antisense 1 promotes HCC invasion, metastasis, and EMT by targeting ZEB1 [83]. A similar regulation mechanism has also been found between ZEB2 and ZEB2 antisense 1 [95]. Recent studies showed that lncRNAs competitively bind with miRNAs that maintain the expression of ZEB1 and EMT, including lncRNA MALAT1 and miR-143-3p, lncRNA TUG1, and Mir-142-3p [86,88].

## 7. Homeobox Genes and Immunotolerance in HCC

Liver sinusoids are involved in the central immunological functions of the liver [123]. With this system, the liver has the capacity to remove different microbes, microbe-associated molecules, and DAMPs, which continuously circulate from the gut to the liver. The diverse innate and adaptive immune cells residing in the liver to detect and clear bloodborne infections include Kupffer, natural killer, natural killer T, and CD4+/8+ T cells. These immune cells respond to large and diverse cell-surface ligands expressed by infected, damaged, or transformed cells, leading to the changes in the innate and adaptive immune reactions through the production of different potent cytokines [124]. Sinusoidal endothelial cells, stellate cells, and hepatocytes participate in the maintenance of balance between immunotolerance and immune activation to prevent liver damage from non-pathological or continuous inflammatory stimuli or systemic immunotolerance [125]. Deregulation of the precisely regulated immunological network promotes the development of HCC, possibly owing to chronic infection (e.g., infection with HBV or HCV), fat accumulation (i.e., NASH), or DAMPs generated as a result of toxic liver damage (alcoholic liver disease) [126].

Rare reports focus on Homeobox gene and immune suppression in tumor cells. One example is that of the intestine-specific Homeobox transcription factor (ISX), which has a consensus homeodomain (Figure 1), and is a proto-oncogene involved in HCC development [55,56,57]. Recently, our lab demonstrated that ISX is involved in a positive feedback loop, including inflammation, tryptophan catabolism, and immune suppression. IL-6 induces the transcriptional activation of ISX to promote the production of tryptophan catabolic enzymes tryptophan 2,3-dioxygenase and indoleamine 2,3-dioxygenase 1 in HCC. Both enzymes increase the level of tryptophan catabolite, kynurenine, and aryl hydrocarbon receptor (AhR) and activate the kynurenine/AHR axis. Its activation promotes a positive feedback mechanism to increase ISX associated proliferation, tumorigenesis, and immunotolerance. Apart from the AHR-dependent immunotolerance, overexpression of ISX induced level of genes encoding the immune modulators CD86 (B7-2) and PD-L1 and presented a repressed CD8^+^ T-cell response.

## 8. Homeobox Genes in HBV- and HCV-Associated HCC

Clinical and epidemiological studies have linked chronic hepatic inflammation to the pathogenesis of HCC [127]. The chronic HBV- and HCV-induced inflammation contributes to the development of HCC [128]. Notably, this tumorigenesis was associated with the regulation of numerous Homeobox genes [30]. The Hox-A13 protein is induced in liver stem-like cells and HBV or HCV-infected HCC, but not in hepatocytes and bile duct epithelia [129]. The function of the prospero-related Hox protein 1 in the progression of HCC is debatable [73,130,131,132,133]. However, it represses HBV antigen expression and genome replication through repression of the enhancer II/core promoter, preS1 promoter, and enhancer I/X promoter of HBV [134]. In HBV-infected HCC cells, IL-6 increases IGF1R and result in the stemness-related properties that evaluate the Oct4/Nanog, which confers poor prognosis [69]. Furthermore, the HBx encoded by the HBV X gene has been shown to be associated with HCC development. The HBX with C-terminally truncation causes more malignant HCC by promoting metastasis and tumorigenicity. The reason is that HBx-ΔC1 is involved in the regulation of the properties of liver cancer stem cells by up-regulating the expression of Homeobox and *NANOG* through the stat3 pathway [135]. The regulation of HCV by Homeobox promotes TICs. Overexpression of HCV non-structural protein NS5A and induction Toll-like receptor 4 (*TLR4*) by alcohol-induced endotoxemia in hepatocyte synergistically generates liver damage and tumor development, resulting in the generation of *Nanog*-positive TICs from the mice model [65,136]. Of note, *TLR4*/*Nanog*-dependent TICs are also found in HCC patients. Research has identified a *TLR4*/*Nanog*-mediated activation of YAP1 and IGF2BP3, which are novel molecules responsible for the inhibition of the TGF-β pathway and the development of chemoresistance [102]. Using a high-cholesterol/high-fat diet, it was shown that a higher proportion of hepatocyte-specific NS5A transgenic mice developed liver tumors containing TICs. This was attributed to the activation of *TLR4-Nanog* and pSTAT3 signaling pathways through an exaggerated EMT via the induction of *Twist1* [65].

## 9. Conclusions

Dysregulated expression of Homeobox genes is wildly identified in different aspects of HCC development, including HBV and HCV infection, TICs, EMT, and immunotolerance. Homeobox genes are discovered in both positive and negative regulation in HCC progression; however, transcriptional regulatory networks of Homeobox genes in HCC remain unclear. Integrating single cell RNA sequencing in HCC to build a systemic interaction network of Homeobox genes in HCC is noteworthy. It can provide more information for the cell type specific mechanism of the regulation of Homeobox genes in the tumor microenvironment and for the design of more efficacious therapy of HCC patients.

In recent years, Homeobox genes have been identified at different stages of the progression of HCC; thus, Homeobox genes are the potential therapeutic targets, but some questions remain to be answered. One of the strategies is regulating the expression or stability of Homeobox genes, but more effort is required to identify the cohesive signaling regulation for using a specific inhibitor and activator or to develop the drug for specific targets. Second, the structure of the homeodomain is not suitable for the development of a small molecular drug to block the DNA-binding ability of Homeobox. The conservation of homeodomain increases the difficulties regarding the specificity of drug and needs to be solved by structure identification. Third, it has been known that the expressions of Homeobox genes in HCC patients are diverse; thus, development of a high-throughput screening method for detecting aberrant expressions of Homeobox genes would be valuable to develop a precise medicine tailored to individual needs. Therefore, more and more basic and clinical studies are required to enhance the knowledge regarding Homeobox genes in HCC patients.

## Figures and Tables

**Table 1:** Homeobox genes suppress hepatocellular carcinoma (HCC) progression.

Homeobox Gene	Experiment Model	Function in HCC	Ref.
aristaless-like homeobox 4 (*ALX4*)	cell lines: Huh7, HepG2, and HCCLM3	Overexpression of *ALX4* inhibits the proliferation, invasion, and EMT.	[31]
BARX homeobox 1 (*BARX1*)	cell lines: HepG2, Huh7, Hep3B, SMMC7721, MHCC97L, MHCC97H, HCCLM3, and HCCLM6 mouse model HCC tissues	1. Low expression of *BARX1* correlates with poor prognosis.	[32]
		2. *BARX1* suppresses invasion and metastasis by inhibiting *MGAT5* and *MMP9* transcription.	
BARX homeobox 2 (*BARX2)*	HCC tissues	Low expression of *BARX2* is correlated with tumor metastasis.	[33]
caudal-type homeobox 1 (*CDX1*)	HCC tissues	Low expression of *CDX1* is associated with poor prognosis.	[34]
growth arrest-specific homeobox (*Gax*)	cell lines: HepG2, Huh7, and HCCLM3 HCC tissues	Gax expression inhibits NF-kappa B signal, and its expression negatively regulated by miR-301a.	[35]
hematopoietically expressed homeobox protein (*Hhex*)	cell line: Hepa1-6 mouse model HCC tissues	1. Overexpression of *Hhex* resulted in decreases expression of c-Jun and Bcl2, and increases expression of P53 and Rb.	[36]
		2. *Hhex* expression attenuates tumorigenicity in nude mice.	
homeobox D10 (*HOXD10*)	cell lines: MHCC97H, MHCC97L, and HepG2	HOXD10 is downregulated by miR-224 repression that causes cell migration and invasion.	[37]
NK2 homeobox 8 (*NKX2.8*)	cell lines: PLC and Hep3B HCC tissues	*NKX2.8* expression is downregulated in HCC, and low *NKX2.8* expression is negatively correlated with poor survival in patients.	[38]
NK3 homeobox 1 (*NKX3.1*)	cell lines: SMMC-7721, Li7, Huh7, HCCLM3 MHCC-97L, HCCLY10, PLC5, and SK-Hep1 mouse model HCC tissues	*NKX3.1* suppresses tumor proliferation and invasion by up-regulating Foxo1 expression.	[39]
paired related homeobox 1 (*PRRX1*)	cell lines: Huh7, Hep3B, HepG2, SMMC7721, and PLC5 HCC tissues	1. Hepatic cancer-stem cell properties are disrupted by *PRRX1* overexpression.	[40,41]
		2. *PRRX1* overexpression induces HCC apoptosis via the p53-signaling.	

EMT—epithelial to mesenchymal transition.

**Table 2:** Homeobox genes promote HCC progression.

Homeobox Gene	Experiment Model	Function in HCC	Ref.
caudal-related homeobox 2 (*CDX2*)	cell lines: MHCC97L and Hep3B	CDX2 binds to CDH17 promoter and modulates its expression.	[42]
distal-less homeobox 2 (*DLX2*)	cell lines: Huh7, HepG2, Hep3B, SMMC7721, MHCC97H, and MHCC97L HCC tissues	1. Overexpression of DLX2 in HCC tissues is an indicator of poor prognosis.	[43]
		2. DLX2 increases sorafenib resistance by promoting the ERK pathway and EMT.	
distal-less homeobox 4 (*DLX4*)	cell line: Hep3B HCC tissues	1. DLX4 is up-regulated in HCC tissues.	[44]
		2. miR-122 binds 3′UTR of *DLX4* for down-regulated its expression.	
goosecoid (*GSC*)	cell lines: MHCC97L, MHCC97H, HCCLM3, SMMC7721, Hep3B, and HepG2 HCC tissues	*GSC* expression is associated with metastasis and EMT in patients.	[45]
homeobox HB9 (*HLXB9*)	cell lines: HLE, HLF, Huh7, HepG2, and Hep3B HCC tissues	HLXB9 upregulation is observed in poorly differentiated HCC with a pseudoglandular pattern.	[46]
homeobox A13 (*HOXA13*)	cell lines: SNU-449 and HepG2 HCC tissues	1. High *HOXA13* expression is positively correlated with tumor size, microvascular invasion, pathological grade, tumor capsula status, AFP level, metastasis, and microvessel density.	[47,48]
		2. Overexpression of HOXA13 increases colony formation on soft agar and migration, and reduces sensitivity to sorafenib.	
homeobox B7 (*HOXB7*)	cell lines: SMMC-7721, MHCC97L, MHCC97H, HCCLM3, PLC, HepG2, and Huh7 mouse model HCC tissues	1. High HOXB7 expression is associated with larger tumor size and a higher rate of biliary invasion.	[49,50,51]
		2. HOXB7 promotes c-Myc and Slug expression through AKT activation, resulting in HCC progression.	
		3. HOXB7 promotes proliferation, migration, and invasion through activation of the MAPK/ERK axis.	
homeobox B9 (*HOXB9*)	cell lines: BEL-7402, BEL-7404, BEL-7405, HepG2, Hep3B, and SNU475 HCC tissues	HOXB9 regulates TGF-β1 and ZEB1 signaling to promote EMT and cancer metastasis.	[52,53]
iroquois homeobox3 (*IRX3*)	cell lines: HepG2 and SMMC7721	IRX3 induces proliferation, migration, and invasion, but its expression is repressed by miR-377.	[54]
intestine-specific homeobox (*ISX)*	cell lines: HepG2 and Huh7 mouse model HCC tissues	1. ISX is a regulator in HCC progression as a prognostic and therapeutic target in HCC.	[55,56,57]
		2. Cyclin D1 and E2F1 are downstream target genes of ISX in HCC.	
		3. ISX involves kynurenine–AHR axis and immunosuppression effect of PD-L1 and CTLA-4 for immune escape by HCC.	
NANOG	cell lines: Huh7, MHCC97L, HepG2, and SMMC7221 mouse model HCC tissues	1. NANOG expression is required for TICs of HCC.	[58,59,60,61,62,63,64,65,66,67]
		2. Nanog maintains TICs through the insulin-like growth factor pathway in HCC.	
		3. Nanog promotes EMT through Stat3-dependent Snail activation.	
		4. HCV-NS5A induces TLR4–NANOG axis, promoting the formation of liver TICs.	
POU class 5 homeobox 1 (*POU5F1* or *OCT4*)	cell lines mouse model HCC tissues	OCT4 expression is required for TICs.	[67,68,69]
pre-B-Cell leukemia homeobox 3 (*PBX3*)	cell lines: HepG2 Huh7, QGY-7701, and BEL-7402 Chick model HCC tissues	miR-33a-3p suppresses the cell growth, spreading, and invasion by inhibiting *PBX3* expression.	[70]
prospero-related homeobox 1 (*PROX1*)	cell lines: Hep3B, Huh7, HepG2, BEL-7402, QGY7701, QGY7703, SMCC7721, and MHCC97H mouse model HCC tissues	1. PROX1 is required for hepatocyte migration.	[71,72,73]
		2. High PROX1 expression is associated with poor survival and tumor recurrence of HCC.	
		3. PROX1 promotes HCC metastasis by induction and stabilization of HIF1a.	
		4. MAZ contributes to Prox1 isoform expressions in HCC.	
		5. PROX1 positively regulate HCC proliferation and sorafenib resistance by enhancing β-catenin signaling.	
short stature homeobox 2 (*SHOX2*)	cell lines: HepG2, Huh7, and SMMC772 HCC tissues	SHOX2 gene is associated with poor prognosis	[74]
sineoculis homeobox homolog 1 (*SIX1*)	cell lines: HepG2 HCC tissues	The expression status of SIX1 is associated with the five-year survival rate duration of patients with early stage (I-II) of HCC, but not the advanced stage (III–IV) of HCC	[75]
zinc finger E-box binding homeobox 1/2 (*ZEB1/2*)	cell lines: Huh-7, HepG2, SMMC7721, Hep3B, SNU449, MHCC97H, HCCLM3, BEL-7402 QGY-7701, PLC5, and SK-Hep1 mouse model HCC tissues	ZEB1/2 is a transcription factor as a hub that promotes tumor invasion and metastasis by inducing EMT (detail in the text)	[76,77,78,79,80,81,82,83,84,85,86,87,88,89,90,91,92,93,94,95,96,97]

ERK—extracellular signal-regulated kinase; AFP— alpha-fetoprotein; AKT—protein kinase B; MAPK—mitogen-activated protein kinase; CTLA-4—T-lymphocyte-associated protein 4; TICs—tumor-initiating stem-like cells; TGF-β—transforming growth factor-β; AHR—aryl hydrocarbon receptor; PD-L1—programmed death-ligand 1; MAZ—MYC-associated zinc finger protein; HCV—hepatitis C virus.

## Abbreviations

Hepatocellular carcinoma (HCC)	transforming growth factor-β (TGF-β)
nonalcoholic fatty liver disease (NAFLD)	reactive oxygen species (ROS)
hepatitis B virus (HBV)	Receptor for activated C kinase1 (RACK1)
hepatitis C virus (HCV)	Myeloid cell leukemia-1 (Mcl-1)
HBV X protein (HBx)	Transforming acidic coiled-coil protein 3 (TACC3)
Aflatoxin B1 (AFB1)	GATA transcription factor 5 (GATA5)
tumor necrosis factor-α (TNF-α)	Level of Atonal homolog 8 (ATOH8)
interleukin 6 (IL-6)	basic-helix-loop-helix (bHLH)
programmed death-ligand 1 (PD-L1)	Zinc finger E-box binding Hox 1 (ZEB1)
programmed cell death 1 (PD-1)	MYC-associated zinc finger protein (MAZ)
tumor-initiating stem-like cells (TICs)	microRNAs (miRNAs)
mesenchymal transition (EMT)	long non-coding RNAs (lncRNAs)
epithelial cell adhesion molecule (EpCAM)	Long non-coding RNA activated by TGF-β (lncRNA-ATB)
octamer-binding transcription factor 4 (*Oct4*)	intestine-specific homeobox transcription factor (ISX)
insulin-like growth factor (IGF) 2	aryl hydrocarbon receptor (AhR)
IGF 1 receptor (IGF1R)	Toll-like receptor 4 (*TLR4*)
vascular endothelial growth factor (VEGF)	Damage-associated molecular patterns (DAMPs)
